# Fatty infiltrate and neck muscle volume in individuals with chronic whiplash associated disorders compared to healthy controls – a cross sectional case–control study

**DOI:** 10.1186/s12891-023-06289-x

**Published:** 2023-03-11

**Authors:** Nils Lund, Olof Dahlqvist Leinhard, James M Elliott, Gunnel Peterson, Magnus Borga, Peter Zsigmond, Anette Karlsson, Anneli Peolsson

**Affiliations:** 1 Department of Health, Medicine and Caring Sciences, Unit of Physiotherapy, Linköping University, Linköping, Sweden; 2 Center for Medical Image Science and Visualization (CMIV), Linköping University, Linköping, Sweden; 3 AMRA Medical AB, Linköping, Sweden; 4 Department of Health, Medicine and Caring Sciences, Linköping University, Linköping, Sweden; 5 Faculty of Medicine and Health, School of Health Sciences, Northern Sydney Local Health District, The Kolling Institute, University of Sydney, St Leonards, NSW Australia; 6 Feinberg School of Medicine, Department of Physical Therapy and Human Movement Sciences, Northwestern University, Chicago, IL USA; 7 Centre for Clinical Research Sörmland, Uppsala University, Uppsala, Sweden; 8 Department of Biomedical Engineering, Linköping University, Linköping, Sweden; 9 Department of Neurosurgery and Clinical and Experimental Medicine, Linköping University, Linköping, Sweden; 10 Occupational and Environmental Medicine Center, Department of Health, Medicine and Caring Sciences, Unit of Clinical Medicine, Linköping University, Linköping, Sweden

**Keywords:** WAD, Whiplash injury, Cervical spine, MRI, Fatty infiltration, Muscle volume

## Abstract

**Background:**

The underlying pathophysiological mechanisms of chronic Whiplash Associated Disorders (WAD) are not fully understood. More knowledge of morphology is needed to better understand the disorder, improve diagnostics and treatments. The aim was to investigate dorsal neck muscle volume (MV) and muscle fat infiltration (MFI) in relation to self-reported neck disability among 30 participants with chronic WAD grade II-III compared to 30 matched healthy controls.

**Methods:**

MV and MFI at spinal segments C4 through C7 in both sexes with mild- to moderate chronic WAD (*n* = 20), severe chronic WAD (*n* = 10), and age- and sex matched healthy controls (*n* = 30) was compared. Muscles: trapezius, splenius, semispinalis capitis and semispinalis cervicis were segmented by a blinded assessor and analyzed.

**Results:**

Higher MFI was found in right trapezius (*p* = 0.007, Cohen’s d = 0.9) among participants with severe chronic WAD compared to healthy controls. No other significant difference was found for MFI (*p* = 0.22–0.95) or MV (*p* = 0.20–0.76).

**Conclusions:**

There are quantifiable changes in muscle composition of right trapezius on the side of dominant pain and/or symptoms, among participants with severe chronic WAD. No other statistically significant differences were shown for MFI or MV. These findings add knowledge of the association between MFI, muscle size and self-reported neck disability in chronic WAD.

**Trial registration:**

NA. This is a cross-sectional case–control embedded in a cohort study.

## Background

Whiplash describes the mechanism of injury consisting of acceleration-deceleration mechanisms of energy transfer to the neck, which may lead to a variety of bony- or soft-tissue injuries and subsequent symptoms (Whiplash Associated Disorders, WAD) [1]. With roughly half transitioning into chronicity [2], WAD is associated with high individual and societal costs [3, 4], and limited activities of daily living for those transitioning from acute to chronic WAD [5].

Soft-tissues commonly exposed in whiplash include neck muscles [6–8]. They are at particular risk of muscle strain injury [9, 10]. Neck muscles are essential for proper neck functioning [11, 12] and are influenced by muscle size [13], specifically contractile muscular content [14]. Muscle fat infiltration (MFI) can occur in muscles [15] replacing contractile muscular content, lowering muscle function [16]. Well-functioning neck muscles are important in chronic WAD and is supported by neck specific exercise, primarily targeting the muscles, being one of the few effective treatments [17–23].

Both functional [24–28] and structural changes in (CSA) [29–34] and MFI [29, 33–39] are evident in WAD. Changes of CSA has been investigated in chronic WAD among dorsal neck muscles (interspinales [32], levator scapulae [29], multifidus [29–32], occipital muscles [29, 30, 32], semispinalis capitis [29–31], semispinalis cervicis [30–32], spinalis [29, 32], splenius capitis [29–31], splenius cervicis [29] and trapezius [29, 30]), as well as MFI in (levator scapulae [29], multifidus [33–39], occipital muscles [33, 35, 36], semispinalis capitis [33, 35, 36], semispinalis cervicis [33, 35, 36, 39], splenius capitis [33, 35, 36, 39] and trapezius [35, 36]).

Larger magnitudes of MFI are correlated with higher disability in chronic WAD [34, 38–40]. MFI and disability may, according to a pilot study, be reversed by neck specific exercise [41]. MFI can develop within two weeks following whiplash [38] and is a specific feature of WAD, not occurring in other chronic neck pain [29, 35, 42]. It is possible that MFI i) is associated with inadequate recovery of function, ii) could represent an initial damage involving both peripheral- and central structures of the neck [43], iii) could represent a risk factor for poor recovery [44]. The precise mechanisms underlying the development of MFI are unknown but seem to be a combination of tissue-based and stress-based interactions [45, 46].

Previous studies have investigated MFI and/or CSA in thin slices at different cervical segments and in different dorsal neck muscles [29–39, 42, 43]. Measurements of CSA have shown conflicting results [29–32]. MFI is shown to distribute unequally among- and within different neck muscles [29, 33–40, 42, 43, 47] and sometimes measurements of muscles have been combined [39]. Only two of the studies investigating dorsal neck muscles used a blind assessor [29, 34] and a clear majority of the participants were female [29–39]. Most studies [29, 30, 32–39, 42, 43] specified WAD grade, and exclusively included participants with grade II (neck pain and musculoskeletal findings [1]) but excluded those with severe symptoms [29, 30, 32, 33, 35–39, 42, 43], i.e., neurological signs (WAD grade III [1]) and one study [31] did not specify WAD grade for its’ participants.

There is a need for improving knowledge of the association between MFI, changes in muscle size and disability [42], as well as verifying previous results via volumetric measures [33, 48]. The underlying pathophysiological mechanisms of chronic Whiplash Associated Disorders (WAD) are not fully understood, and more knowledge of morphology is needed to better understand the disorder, improve diagnostics and treatments. The aim was to investigate dorsal neck muscle volume (MV) and fat infiltration (MFI) in relation to self-reported neck disability among participants with chronic WAD grade II-III compared to matched healthy controls.

## Methods

### Study participants

Thirty participants (14 men, 16 women, mean age 41±11) with chronic WAD (>6 month, mean 18±9) and 30 healthy controls, matched for age and sex (14 men, 16 women, mean age 41±11) were recruited. Data was collected year 2012-2013 at a university hospital in the south of Sweden and later processed and analyzed.

Neck Disability Index (NDI): a measure of self-reported disability [49], reliable and valid for participants with neck pain disorders [50], was used to divide chronic WAD participants into a mild- to moderate chronic WAD group (NDI; ≤ 20% to < 40%) and a severe chronic WAD group (NDI; ≥ 40%) [34]. For demographic details, see Table 1.

**Table 1:** Demographic details of participants divided into three groups according to Neck Disability Index (NDI) [51]. Data are mean ± SD

Group	n	Age (years)	Sex	BMI (kg/m^2^)	Time since injury (months)	NDI (%)	VAS
Healthy controls	30	41 ± 11	16 F, 14 M	24 ± 3	-	-	-
Mild/moderate chronic WAD (NDI; ≤ 20%– < 40%)	20	39 ± 11	10 F, 10 M	25 ± 4	20 ± 10	27 ± 7	30 ± 5
Severe chronic WAD (NDI; ≥ 40%)	10	46 ± 9	6 F, 4 M	26 ± 3	16 ± 8	52 ± 11	53 ± 5

The chronic WAD group was consecutively recruited to the current exploratory cross sectional case-control study from an ongoing randomized clinical trial (RCT) [17, 52] with inclusion criteria: 18-63 years of age, presenting with chronic WAD grade II (clinical musculoskeletal findings emanating from the neck) or III (as grade II with additional neurological findings [1]) verified in a clinical examination following a whiplash injury six months to three years prior to inclusion, NDI >20% and/or VAS > 20 mm and being right-handed with dominant right sided symptoms. Exclusion criteria for the chronic WAD group were known or suspected serious physical pathology including myelopathy, spinal tumor, spinal infection, or ongoing malignancy, contraindications for magnetic resonance imaging (MRI) such as metallic implants, claustrophobia and pregnancy, spinal fracture or subluxation, earlier neck trauma with persistent symptoms, cervical spine surgery, neck pain that caused a >1 month absence from work in the year prior to the WAD trauma, signs of traumatic brain injury at inclusion, generalized or dominant pain elsewhere in the body, diseases or other injuries that might prevent full study participation, diagnosis of a severe psychiatric disorder, known drug abuse and inability to answer the Swedish questionnaires [52].

Inclusion criteria for the healthy control group were right-handed, age- and sex matched to the participants with chronic WAD and exclusion criteria were contraindications for MRI, present or past neck pain, dysfunction, or related disability, rheumatological or neurological disease/conditions, generalized myalgia and history of neck trauma, neck pain or lower back pain. The healthy control group was recruited from a convenience sample of university and hospital staff as well as researchers’ acquaintances.

### MRI protocol

Magnetic resonance images were acquired with a Philips Ingenia 3T scanner (Philips Health Care, Best, The Netherlands) using the built-in phased-array posterior coil, a 32-channel head coil, and an anterior flexible coil placed adjacent to the head coil. The participants were imaged in the supine, headfirst position. A 3D dual-echo gradient-echo sequence was used with out-of-phase and in-phase echo times of 3.66 ms and 7.24 ms, respectively. The echo times were chosen to enable the production of high-resolution images. The repetition time was 10 ms and the flip angle was 10° with a total acquisition time of 9 minutes. The images included cervical segmental levels C4 through C7 and were angled so that the in-plane images were parallel to the cervical segments and perpendicular to the long axis of the cervical musculature. The acquired image resolution was 0.75 × 0.75 × 0.75 mm^3^. Phase-sensitive reconstruction was used to acquire fat- and water- separated images [53, 54].

### Image analysis

**Fig. 1:**
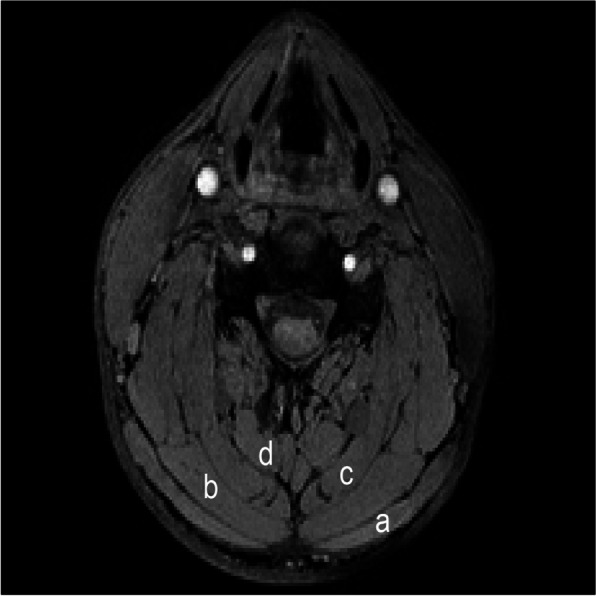
Viewed in axial plane (X–Y) at C5 level using the water-only image. **a** trapezius, **b** splenius, **c** semispinalis capitis and **d** semispinalis cervicis

**Fig. 2:**
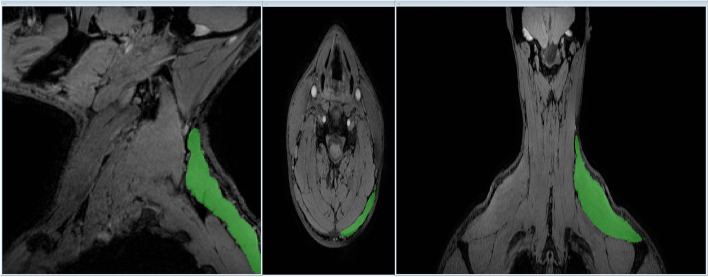
Segmentation of left trapezius. Views from left to right; sagittal plane (Y–Z), axial plane at C5 (X–Y) and coronal plane (X–Z)

Trapezius, splenius, semispinalis capitis and semispinalis cervicis (figure 1) were semi-automatically segmented via Image Foresting Transform (IFT) [55] between spinal segmental levels C4 to C7. Semi-automatic segmentation was performed by an assessor (N.L.), blinded to group allocation, defining each individual muscle in the horizontal-, frontal- and sagittal plane creating a 3D mask used for analysis. The assessor used one set of markers for the muscle of interest and another set of markers for other tissues of no interest for the current study. IFT uses the different markers to continuously suggest regions of interest and disinterest of which the assessor can further define, resulting in a complete 3D model of the muscle without addition of surrounding tissue (Figure 2).

The interrater reliability for the image analysis performed by (N.L.) was determined by presenting 18 of the participants’ images twice in random order and calculated using Two-way random absolute agreement single rater - intraclass correlation coefficient (ICC) [56] with an ICC of 0.91-0.99 for the MV of the muscles segmented. Image analysis was double checked with a second blinded rater (JM), a professor and musculoskeletal physical therapist with >15 years’ experience of MRI analysis.

MV was calculated by summating all pixels within the segmented mask multiplied with the image resolution. The fat infiltration was estimated via MATLAB 2017b using fat fraction, in which the amount of fat signal within the muscle divided by the sum of the water and fat signal within the muscle: fat fraction = fat/ (fat + water).

### Statistical analysis

All statistical analyses were performed using IBM SPSS version 28 (IBM Corporation, Armonk, NY). Normal distributions were investigated using histograms and Shapiro-Wilk test for age, BMI, time since injury, NDI and for MV and MFI. Normal distribution could not be assumed for most variables, resulting in the use of non-parametric testing; Kruskal-Wallis H Test, and if statistically significant, followed by a post-hoc Mann-Whitney U-test. The significance level was set to p<0.05. Cohen’s d was computed to represent the magnitude of the effect size (ES) of statistically significant between group differences.

## Results

**Fig. 3:**
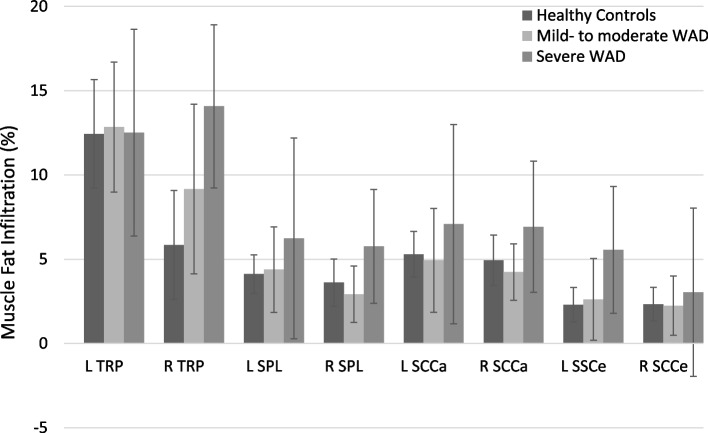
Muscle fat infiltration is expressed as percentages (%) with confidence interval set to 0.95. Abbreviations: HC, healthy controls; WAD, Whiplash Associated Disorders; L, left; R, right; TRP, trapezius; SPL, splenius; SSCa, semispinalis capitis; SSCe, semispinalis cervicis

**Fig. 4:**
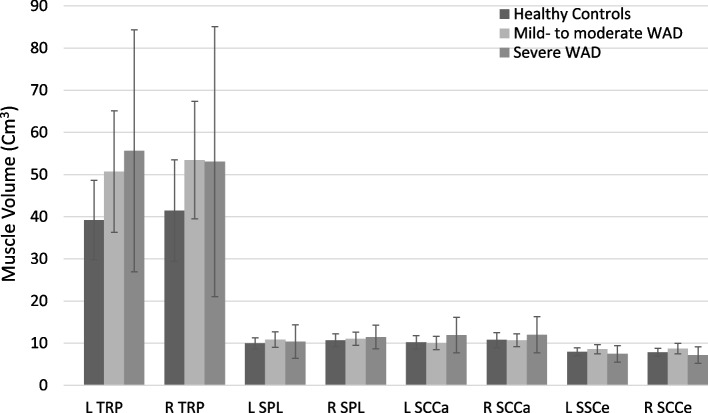
Muscle volume are expressed as cm^3^ with confidence interval set to 0.95. Abbreviations: HC, healthy controls; WAD, Whiplash Associated Disorders; L, left; R, right; TRP, trapezius; SPL, splenius; SSCa, semispinalis capitis; SSCe, semispinalis cervicis

A statistically significant difference in MFI was seen between groups for right trapezius (*p*=0.03, ES = 0.6), with higher MFI in severe WAD group compared to healthy controls (*p*=0.007, ES = 0.9). No statistically significant difference was seen between healthy controls and the mild- to moderate chronic WAD group (*p*=0.32) or between the two chronic WAD groups (*p*=0.10). No other statistically significant differences between groups were obtained for MFI (*p*=0.22-0.95) (Table 2) or MV (*p*=0.20-0.76) (Table 3). Severe chronic WAD show consistently higher MFI compared to healthy controls and mild- to moderate chronic WAD for all muscles except left trapezius (Figure 3). Left and right trapezius show increased MV in the chronic WAD groups compared to the healthy control group (Figure 4).

**Table 2:** Median values ± interquartile range for muscle fat infiltration (%)

Muscles	HC	MM WAD	S WAD	Sig
L TRP	11 ± 14	12 ± 12	11 ± 14	0.95
R TRP	3 ± 14	7 ± 14	14 ± 12	0.03^*^
L SPL	4 ± 3	2 ± 9	6 ± 11	0.63
R SPL	3 ± 3	2 ± 6	6 ± 9	0.22
L SSCa	5 ± 5	3 ± 9	6 ± 11	0.62
R SSCa	4 ± 4	4 ± 5	7 ± 9	0.38
L SSCe	2 ± 3	2 ± 6	5 ± 10	0.27
R SSCe	2 ± 3	2 ± 6	4 ± 6	0.5

Abbreviations: *HC* healthy controls, *WAD* Whiplash Associated Disorders, Sig., Kruskal Wallis H-test significance, *L* left, *R* right, *TRP* trapezius, *SPL* splenius, *SSCa* semispinalis capitis, *SSCe* semispinalis cervicis

^*^ Statistically significant

**Table 3:** Median values ± interquartile range for muscle volume (cm^3^)

Muscles	HC	MM WAD	S WAD	Sig
L TRP	34 ± 39	39 ± 32	43 ± 71	0.31
R TRP	32 ± 50	47 ± 42	42 ± 71	0.23
L SPL	9 ± 6	10 ± 5	9 ± 10	0.58
R SPL	9 ± 7	11 ± 6	9 ± 7	0.76
L SSCa	9 ± 8	10 ± 6	9 ± 11	0.67
R SSCa	10 ± 8	10 ± 5	10 ± 11	0.75
L SSCe	7 ± 4	8 ± 3	7 ± 5	0.33
R SSCe	7 ± 4	8 ± 3	7 ± 3	0.2

Abbreviations: *HC* healthy controls, *WAD* Whiplash Associated Disorders, Sig., Kruskal Wallis H-test significance, *L* left, *R* right, *TRP* trapezius, *SPL* splenius, *SSCa* semispinalis capitis, *SSCe*, semispinalis cervicis

## Discussion

The main finding is a statistically significantly higher MFI in the right trapezius, the side of dominant pain and/or symptoms, among participants with severe chronic WAD compared to healthy controls. This may be explained by Trapezius being exposed to large muscle strain [10] and injury [57] due to rapid activation of Trapezius in whiplash [6]. Higher MFI in Trapezius implies reduced functional muscle mass [14] and function [15] in a muscle important for neck movement [11].

Like the main finding, albeit only by a statistically non-significant trend, MFI is higher in splenius capitis, semispinalis capitis and semispinalis cervicis among participants with severe chronic WAD with highest MFI on the right side. This may be due to a too small sample size and may be a limitation of the study.

The main finding and statistically non-significant trends align with previous studies showing higher MFI in chronic WAD compared to healthy controls [29, 36]. The current study also aligns with previous work [39] showing no statistically significant difference in MFI for splenius capitis combined with sternocleidomastoid, and a statistically non-significant trend for higher MFI in participants with chronic WAD. However, the comparison is difficult considering the measurement methods used in this study (single muscles right and left) did not combine certain muscles (multifidus and semispinalis cervicis) [39].

The current study's statistically non-significant trend towards higher MFI in the superficial musculature i.e., trapezius and consistently less MFI in the deeper lying musculature contrasts earlier findings of higher MFI in deeper lying neck musculature as shown in anterior neck musculature [47] and dorsal deep- compared to superficial musculature [39].

The current study, showing no statistical differences between groups regarding MV, aligns with previous studies reporting unchanged CSA of splenius capitis [29–31], semispinalis capitis and semispinalis cervicis [31]; but is unsupportive of both lower CSA of trapezius and semispinalis capitis among WAD participants in another study [29]. There is a statistically non-significant trend for higher MV in the left and right trapezius among chronic WAD participants compared to healthy controls, seemingly independent of handedness and right sided dominant pain. The similarities regarding MV in the left and right trapezius, and in relation to the higher MFI being shown in the right trapezius, further highlights the importance of MFI measurements in conjunction with MV.

These findings improve knowledge of the association between MFI, muscle size and self-reported neck disability in chronic WAD. Implications can be made for exercising the most painful and/or symptomatic side, focusing on Trapezius as well as persons with high degree of disability; potentially reversing MFI [41] and reducing disability [17, 41]. However, this needs to be further evaluated in longitudinal intervention studies.

In the current study MFI is likely to have had the time to develop as it is seen within two weeks following a whiplash injury [38] and, with mean time post injury being 20 and 16 months for the two WAD groups respectively, comparable to another study showing higher MFI at a mean 20-month post injury [36]. Higher MFI in WAD participants have also been shown at 12 months post injury [39] and at 7 years post injury [29]. Given the severity of WAD, the presence of high MFI is not surprising, and this is supported by previous studies with WAD II [29, 36, 39]. Furthermore, this same cohort demonstrated higher MFI in the severe WAD group in multifidus [34]. Although higher MFI being shown in upper cervical segmental levels [47], the levels above C4 not included in the current study are unlikely affecting the result to a significant degree due to the low relative muscle volume compared to the volume analyzed (C4-C7).

The current study is unique of including participants with WAD-related neurological findings (WAD III) in relation to previous studies on the same muscles involving exclusively musculoskeletal findings (WAD II) [29, 30, 32, 33, 35–39, 42, 43]. It is unlikely that inclusion of WAD III attenuates MFI in the current cohort, rather it hypothetically should increase the chance of MFI findings, supported by MFI being shown in multifidus among the WAD participants [34] and greater MFI being associated with higher severity of WAD [29, 35–37, 43, 47].

Keeping demographic details known to affect MV and possibly MFI as equal as possible between groups using age- and sex-matched controls minimizes the number of uncontrolled factors influencing the results. Age [58], sex [59] and BMI [60] have all been shown to influence MV and are in the current study consistent between all groups. Including both men and women increases the standard deviation of MV due to the relative muscle size difference of men and women [59] and likely contribute to larger muscle sizes as compared to previous studies based predominantly on females [29–34]. The relatively high degree of men in the mild- to moderate WAD group might inflate the MV in comparison to both the severe WAD group and the control group; a potential contributor to the relatively high MV found in mild- to moderate group compared to the other groups.

The differences observed in this study compared to previous studies could be due to numerous factors. One of them being unequal distribution of MFI within- and among different neck muscles [29, 33–40, 42, 43, 47], causing a varying degree of MFI being measured via MV and CSA respectively, due to variance in specific spatial distribution. Other potential factors include the use of different MRI machines [61] and segmentation software [62]. A known factor in the current study is a risk of T1-bias for the MFI measurements, potentially inflating the absolute value of MFI but without affecting between group differences.

A limitation of the study is a lack of power and sample size calculation. With the number of research participants being similar to several other studies eliciting statistically significant differences between groups [29, 31, 34] it is possible that an even higher number, in line with some previous studies [36, 37] and a more equal distribution between groups, could have strengthened the current study’s results.

## Conclusion

There are quantifiable changes in muscle composition of right trapezius on the side of dominant pain and/or symptoms, among participants with severe chronic WAD. No other statistically significant differences were shown for MFI or MV. These findings add knowledge of the association between MFI, muscle size and self-reported neck disability in chronic WAD.

## Data Availability

Data are available from the corresponding author upon reasonable request and after ethical permissions.

## Abbreviations

BMI: Body mass index
CSA: Cross-sectional area
ICC: Intraclass correlation coefficient
IFT: Image foresting transform
MFI: Muscle fat infiltration
MRI: Magnetic resonance imaging
MV: Muscle volume
NDI: Neck disability index
RCT: Randomized clinical trial
SPL: Splenius muscle
SSCa: Semispinalis capitis muscle
SSCe: Semispinalis cervicis muscle
TRP: Trapezius muscle
VAS: Visual analogue scale
WAD: Chronic whiplash associated disorders
